# Preparation of Attapulgite/CoFe_2_O_4_ Magnetic Composites for Efficient Adsorption of Tannic Acid from Aqueous Solution

**DOI:** 10.3390/ijerph16122187

**Published:** 2019-06-20

**Authors:** Yue Teng, Zhiying Liu, Ke Yao, Wenbin Song, Yongjun Sun, Hailing Wang, Yanhua Xu

**Affiliations:** 1 College of Environmental Science and Engineering, Nanjing Tech University, Nanjing 211800, China; sunadnmoon19960203@163.com (Y.T.); zhiying555@njtech.edu.cn (Z.L.); yaoke214421@163.com (K.Y.); swb19960311@163.com (W.S.); 2 College of Urban Construction, Nanjing Tech University, Nanjing 211800, China; sunyongjun@njtech.edu.cn

**Keywords:** attapulgite, adsorption, CoFe_2_O_4_ magnetic nanoparticle, tannic acid

## Abstract

The use of attapulgite (ATP)-based materials for adsorption of pollutants from water and wastewater has received growing attention. However, recovering ATP-based adsorbents remains a challenge. In this study, a magnetic adsorbent ATP/CoFe_2_O_4_ with high tannic acid (TA) adsorptive capacity was fabricated via a facile co-precipitation approach and was well characterized. The loaded CoFe_2_O_4_ particles were embedded into the adsorbent surfaces to allow magnetic separability. For this material, its TA adsorption kinetics, isotherm behavior, and magnetic separation efficiency are reported. The developed magnetic composites had rapid sorption kinetics of 3 h, high sorption capacity of 109.36 mg/g, and good magnetic separation efficiency of 80%. The used ATP/CoFe_2_O_4_ was successfully regenerated by NaOH and reused five times without a substantial reduction in TA removal and magnetic performance. Intermolecular hydrogen bonding formation and surface complexation were identified as the sorption mechanisms of TA by ATP/CoFe_2_O_4_.

## 1. Introduction

Tannic acid (TA) is a water-soluble polyphenolic organic pollutant with an average molecular weight of 1702 [1]. It is commonly present in wastewater from the medicinal plant, paper, and leather industries [2]. TA is a precursor of carcinogenic disinfection byproducts in the drinking water supply and poses a serious toxicological threat to the ecosystem and human health [3]. Effective techniques to remove TA from wastewater must be developed. 

Thus far, various treatment technologies have been developed, and adsorption has elicited growing interest because of its high efficiency [4], simple design [5], flexible operation, and easy scale-up [6]. Different kinds of adsorbents have been explored such as activated carbon, zeolite, chitosan, and resins [7,8]. Activated carbons offer an attractive option for efficient removal of organic contaminants from water. However, they have limited success with regard to the removal of bulky organic pollutants, such as TA, due to size-exclusion and pore-blockage effects [9]. In addition, the large-scale and practical use of activated carbons is limited by engineering difficulties because of the high cost [10]. To address this issue, many low-cost adsorbents, such as attapulgite (ATP), have been developed for TA removal. ATP is a kind of silicate clay mineral rich in aluminum, magnesium, and other elements. Owing to its unique chain layer structure, ATP has several advantages, which are its large specific surface area, excellent adsorption performance, low cost, and abundant supply [11]. Powdered ATP is easily hydrolyzed though and does not precipitate rapidly after adsorption [12]. Therefore, industrial sludge causes secondary pollution [13]. Magnetic separation is faster and more effective than traditional methods for separating particles from wastewater [14]. 

In the past few years, ATP-based magnetic particles have been proposed as adsorbents for environmental decontamination [15], and they exhibit a high adsorption capacity due to their superparamagnetic nature and unique physical and chemical properties, such as high dispersibility and relatively large surface area [16,17]. Tang et al. reported a facile and green hydrothermal approach to fabricate magnetic carboxyl-functionalized ATP/carbon nanocomposites on the basis of spent bleaching earth. The adsorbent can be easily recycled due to its magnetism, and its adsorption capacity slightly decreases after six adsorption–desorption cycles [18]. Lu et al. prepared ATP–iron oxide magnetic composites via coprecipitation. The composites have a saturation magnetization value of 32.7 emu/g, and they can be easily separated from aqueous solution by using large-scale magnetic separation [19].

In this work, ATP/CoFe_2_O_4_ magnetic composites were synthesized using chemical coprecipitation for TA removal and were characterized via scanning electron microscopy (SEM), energy dispersive X-ray spectroscopy (EDX), Fourier transform infrared spectroscopy (FTIR), powder X-ray diffractometry (XRD), and vibrating sample magnetometry (VSM). The adsorption performance of ATP/CoFe_2_O_4_ was evaluated through kinetic and isothermic analyses. The effects of the number of cycles on adsorption capacity and magnetic separation performance were also investigated. Moreover, the underlying adsorption mechanisms were elucidated using FTIR.

## 2. Materials and Methods 

### 2.1. Materials

ATP was supplied by Jiuchuan Clay Technology Co., Ltd. (Nanjing, Jiangsu, China). Fe(NO_3_)_3_·9H_2_O (iron (III) nitrate nonahydrate) and Co(NO_3_)_2_·6H_2_O (cobalt (II) nitrate hexahydrate) were purchased from Sinopharm Chemical Reagent Co., Ltd. (Shanghai, China). TA (C_76_H_52_O_46_, molecular weight = 1702 per mole) was purchased from Aladdin Industrial Corporation (Southern California, USA). NaOH was obtained from Nanjing Chemical Reagent Co. Ltd. (Nanjing, Jiangsu, China). All chemicals and reagents were of analytical grade and used as received without purification. 

### 2.2. Synthesis of ATP/CoFe_2_O_4_ Magnetic Composites

ATP/CoFe_2_O_4_ magnetic composites were prepared via coprecipitation. First, 8.08 g of Fe(NO_3_)_3_·9H_2_O and 2.909 g of Co(NO_3_)_2_·6H_2_O were dissolved in 400 mL distilled water. Second, 2.35 g of attapulgite was poured into the solution with constant stirring for 10 min. Third, 40% (w/w) sodium hydroxide solution was added to the mixture until the pH rose to 11. Then, the mixture was warmed up to 75 °C and refluxed for 3 h at low temperature. Finally, ATP/CoFe_2_O_4_ was separated under the condition of an applied magnetic field and dried at 65 °C in a vacuum oven for 12 h. In accordance with ATP/CoFe_2_O_4_ preparation, CoFe_2_O_4_ magnetic nanoparticles were synthesized without adding ATP.

### 2.3. Characterization of the Magnetic Sorbent

The microscopic surface morphology of ATP/CoFe_2_O_4_ was monitored using SEM (S-3400N II, Hitachi, Marunouchi, Japan). The elemental composition of samples was characterized using an EX-250 Spectrometer (S-3400N II, Hitachi, Marunouchi, Japan). The surface functional groups were determined using FTIR (IRAffinity-1, SHIMADZU Co., Ltd., Shanghai, China). FTIR analysis was performed in the range of 400–4000 cm^−1^ using KBr pellets. XRD studies were conducted with Cu Kα (λ = 0.154 nm) radiation (X’TRA, ARL, Ecublens, Switzerland). The magnetic properties of ATP/CoFe_2_O_4_ and CoFe_2_O_4_ were determined using VSM (WKO-2T, Beijing Wuke Optoelectronic Technology Co., Ltd., Beijing, China).

### 2.4. Batch Adsorption Experiments

An investigation of the adsorption kinetics of TA on the as-prepared ATP/CoFe_2_O_4_ magnetic composites was conducted by mixing 1 g of adsorbents with 1 L of 200 mg/L TA solution at 298 K to determine the time needed to reach adsorption equilibrium. Samples were drawn periodically for TA concentration analysis [20]. The adsorption isotherm experiments were performed at 298 K, 308 K, and 318 K. ATP/CoFe_2_O_4_ (0.1 g) was dispersed in 100 mL of 25, 50, 100, 150, or 200 mg/L TA solution. The magnetic sorbent (0.1 g) was suspended in 100 mL of TA solution with an initial concentration of 200 mg/L to evaluate the reusability of ATP/CoFe_2_O_4_. After mixing for 5 h at 298 K, ATP/CoFe_2_O_4_ was magnetically separated and then suspended in 8% (w/w) NaOH solution for 1 h. The regenerated ATP/CoFe_2_O_4_ was washed with distilled water to remove the remaining NaOH solution and dried for the next cycle. The adsorbent was placed in an Erlenmeyer flask, shaken at room temperature for 30 min, and poured into the cuvette of the turbidimeter. The turbidity meter reading was the turbidity of the sample when it was completely dispersed in distilled water. Subsequently, a strong magnet was placed inside the cuvette obtained from the turbidimeter. The turbidities at different time intervals were determined to calculate the magnetic separation rate.

### 2.5. Analysis 

The TA concentration was determined using a UV/vis spectrometer (Lambda 25, Perkin-Elmer, Shanghai, China). The TA equilibrium adsorption capacity was calculated as follows:

(1) $$Q_{e}=\frac{V\left(C_{e}-C_{0}\right)}{m},$$

where *V* is the solution volume (L); *C*_0_ and *C*_e_ are the initial concentration and the equilibrium adsorption concentration (mg/L), respectively; and *m* is the adsorbent mass (g).

The adsorption kinetics was analyzed using the quasi-first-order and quasi-second-order models by using Equations (2) and (3) as follows:

(2) $$\ln\left(q_{e1}-q_{t}\right)=\ln q_{e1}-k_{1}t,$$

(3) $$\frac{t}{q_{t}}=\frac{1}{k_{2}q_{e2}^{2}}+\frac{t}{q_{e2}},$$

where *q*_e_ (mg/g) is the adsorption capacity at equilibrium and *q*_t_ (mg/g) is the adsorption at time t; k_1_ g/(mg.min) and k_2_ are the quasi-first- and quasi-second-order rate constant, respectively.

The experimental adsorption data were fitted using Langmuir isotherm and Freundlich isotherm models. The linear expressions of the isotherm equations of Langmuir (Equation (4)) and Freundlich (Equation (5)) are as follows [21]:

(4) $$\frac{C_{e}}{q_{e}}=\frac{1}{bq_{m}}+\frac{C_{e}}{q_{m}},$$

(5) $$\ln\;q_{e}=\ln\;k+\frac{1}{n}\ln\;C_{e},$$

where *C*_e_ (mg/L) and *q*_e_ (mg/g) are the concentration of TA and adsorption capacity at the adsorption equilibrium state, respectively; *q*_m_ (mg/g) is the maximum adsorption capacity corresponding to the monolayer coverage on the surface of the absorbent; *b* (L/mg) is the Langmuir isotherm coefficient; and *n* and *k* are the Freundlich isotherm constants.

The thermodynamic parameters (*G*) (kJ/mol), enthalpy change (*H*) (kJ/mol), and entropy change (*S*) (J/(mol·K)) were calculated from the following Gibbs equations:

(6) ΔG=ΔH−TΔS,

(7) ΔG=−RTln k,

where *R* is the gas constant (8.314 J/(K·mol)), *T* is the absolute temperature (K), and *k* is the equilibrium constant (m/M).

The desorption capacity and desorption rate were calculated according to the following two formulas:

(8) $$Q_{\mathrm{de}}=\frac{C_{\mathrm{de}1}V_{1}-C_{\mathrm{de}2}V_{2}}{m},$$

(9) $$\eta =\frac{Q_{\mathrm{de}}}{Q_{\mathrm{ad}}},$$

where *Q*_de_ is the desorption capacity (mg/g), *C*_de1_ (mg/L) is the TA concentration in alkali-eluting solution, and *C*_de2_ is the TA concentration in water-eluting solution. *V*_1_ and *V*_2_ (mL) represent the volume of NaOH and distilled water used, respectively, and *m* is the adsorbent dosage (g). η is the desorption rate (%) and Q_ad_ is the adsorption capacity (mg/g).

The calculation equation of the magnetic separation rate is as follows:

(10) $$S=\left(1-\frac{T_{i}}{T-T_{0}}\right)\times 100\%,$$

where *T*_i_ is the turbidity of the mixture at different points in the magnetic field, *T* is the turbidity when the adsorbent is completely dispersed, and *T*_0_ is the turbidity of the distilled water.

## 3. Results and Discussion

### 3.1. Characterization 

#### 3.1.1. SEM and EDX

**Figure 1:**
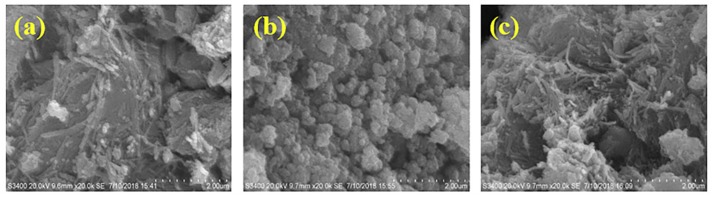
SEM images of attapulgite (ATP) (**a**), CoFe_2_O_4_ (**b**), and ATP/CoFe_2_O_4_ (**c**). SEM: Scanning electron microscopy.

Figure 1 shows the surface morphology of ATP, CoFe_2_O_4_, and ATP/CoFe_2_O_4_ characterized using SEM. Figure 1a is the SEM image of ATP, which is rodlike with a length and width of approximately 100 and 40 nm, respectively. The microstructure of ATP is mainly a needle-like cluster with disordered accumulation and some lamellar mineral particles. These needle-shaped agglomerates contribute to the large specific surface area of attapulgite, with a large adsorption capacity for TA. The SEM image in Figure 1b reveals that the CoFe_2_O_4_ nanoparticles have an almost spherical structural morphology. However, the particle size is nonuniform, and the sample is composed of small discrete particles and several multigrain agglomerations. The average particle size of the synthesized CoFe_2_O_4_ is approximately 100 nm. The agglomeration of nanoparticles observed from the SEM micrograph is expected because they experience a permanent magnetic moment proportional to their volume [22,23]. The SEM image (Figure 1c) of ATP/CoFe_2_O_4_ shows a cubic-like nanostructure. The number of pores in the ATP/CoFe_2_O_4_ magnetic composites is reduced compared with that in ATP, but the ATP crystal is still visible and CoFe_2_O_4_ is randomly distributed on the rod-shaped particles of ATP, indicating that CoFe_2_O_4_ nanoparticles are attached to ATP. The results of EDX elemental analysis of CoFe_2_O_4_ nanoparticles and ATP/CoFe_2_O_4_ are shown in Table 1. CoFe_2_O_4_ magnetic nanoparticles and ATP/CoFe_2_O_4_ are mainly composed of three elements: C, O, and Fe. Na is present in both particles given that NaOH solution is used during preparation to adjust the pH and create an alkaline environment.

#### 3.1.2. FTIR

**Figure 2:**
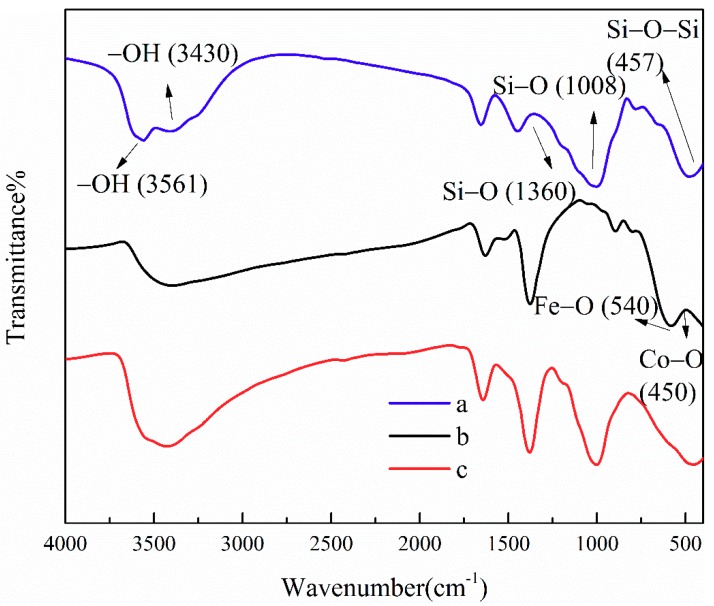
FTIR spectra of ATP (**a**), CoFe_2_O_4_ (**b**), and ATP/CoFe_2_O_4_ magnetic composites (**c**). FTIR: Fourier transform infrared spectroscopy.

Figure 2 shows the FTIR spectra of ATP, CoFe_2_O_4_, and ATP/CoFe_2_O_4_. The absorption bands at 3561 and 3430 cm^−1^ of the FTIR spectrum are assigned to the O–H stretching vibrations of structural water and other water molecules (i.e., zeolitic water and superficially adsorbed water in APT). The absorption band at 457 cm^−1^ corresponds to the Si–O–Si groups of the tetrahedral sheets, and 1008 and 1360 cm^−1^ are attributed to the Si–O stretching vibration. The adsorption peaks at 540 and 450 cm^−1^ are assigned to tetrahedral metal stretching (Fe–O) and octahedral metal stretching (Co–O) and indicate the spinel ferrite structure form (Figure 2b) [24]. The adsorption peak at approximately 3500 cm^−1^ associated with the stretching vibration of O–H appears in the above three samples [25]. This peak is slightly shifted in the ATP/CoFe_2_O_4_, which suggests that CoFe_2_O_4_ is present in ATP/CoFe_2_O_4_; also, the CoFe_2_O_4_ nanoparticles were successfully attached to ATP/CoFe_2_O_4_. The characteristic absorption peaks of ATP and ATP/CoFe_2_O_4_ are substantially the same, but their intensities are slightly changed, indicating that CoFe_2_O_4_ particles do not have a remarkable influence on the chemical composition and properties of ATP (Figure 2a,b). 

#### 3.1.3. XRD 

**Figure 3:**
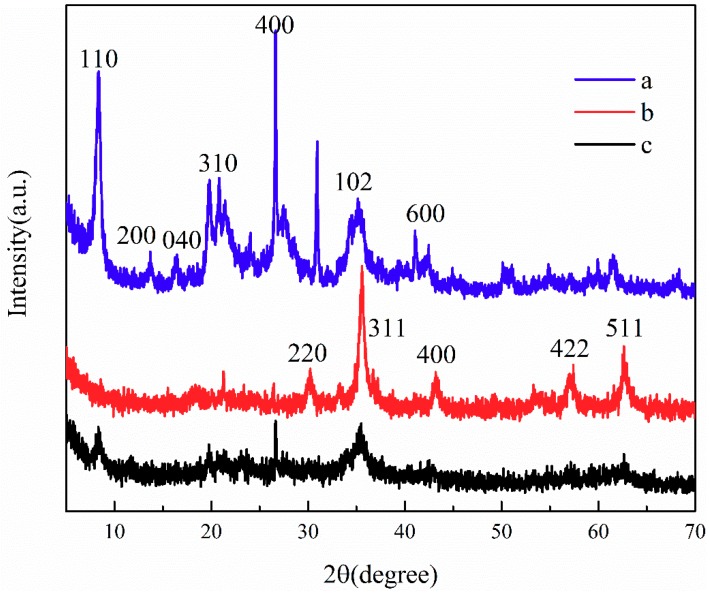
XRD patterns of ATP (**a**), CoFe_2_O_4_ (**b**), and ATP/CoFe_2_O_4_ magnetic composites (**c**).

To determine the crystal phase of the nanoparticles, the sample was analyzed using XRD in the 2θ region of 5–70°. Figure 3 shows the standard XRD patterns of ATP, CoFe_2_O_4_, and ATP/CoFe_2_O_4_. As shown in Figure 3a, the XRD pattern of APT exhibits main characteristic signs at 2θ = 8.4°, 13.7°, 19.8°, 21.5°, 27.5°, 34.7°, and 42.5°(Joint Committee on Powder Diffraction Standards (JCPDS)) card no.2120958), which correspond to the (110), (200), (040), (310), (400), (102), and (600) planes, respectively. The sharper the diffraction peak, the higher the degree of crystallization of the sample [26]. The intensity of the characteristic diffraction peak at 2θ = 8.38° and 26.64° is large, and the diffraction peak is sharp, indicating that the crystal phase of the ATP used is relatively complete. The XRD pattern confirms that CoFe_2_O_4_ formed with space group Fd-3m and a spinel structure [27]. The sharp diffraction peak indicates that the crystal phase of CoFe_2_O_4_ is relatively complete. The diffraction peaks around 29.42°, 35.58°, 42.82°, 52.12°, and 61.64° are attributed to the reflections of the (220), (311), (400), (422), and (511) planes of CoFe_2_O_4_, respectively (JCPDS card no.22-1086) [28]. The average crystallite size of CoFe_2_O_4_ calculated using Scherrer’s formula was approximately 20 nm. The (311) plane was chosen to calculate the crystallite size of CoFe_2_O_4_ nanoparticles. Almost all the peaks of ATP/CoFe_2_O_4_ may be assigned to the spinel structure of CoFe_2_O_4_. The observed diffraction peak at 2θ = 35.58° was of the CoFe_2_O_4_ (311) plane, confirming that the CoFe_2_O_4_ nanoparticles were successfully attached to ATP/CoFe_2_O_4_ [29]. When compared with the XRD pattern of the ATP, the XRD pattern of ATP/CoFe_2_O_4_ suggests that the characteristic diffraction peaks of the attapulgite do not shift, but their strength decreases, indicating that the ATP/CoFe_2_O_4_ preserves the crystal structure of the attapulgite well and that the crystal structure of the ATP suffers little damage during preparation.

#### 3.1.4. VSM

**Figure 4:**
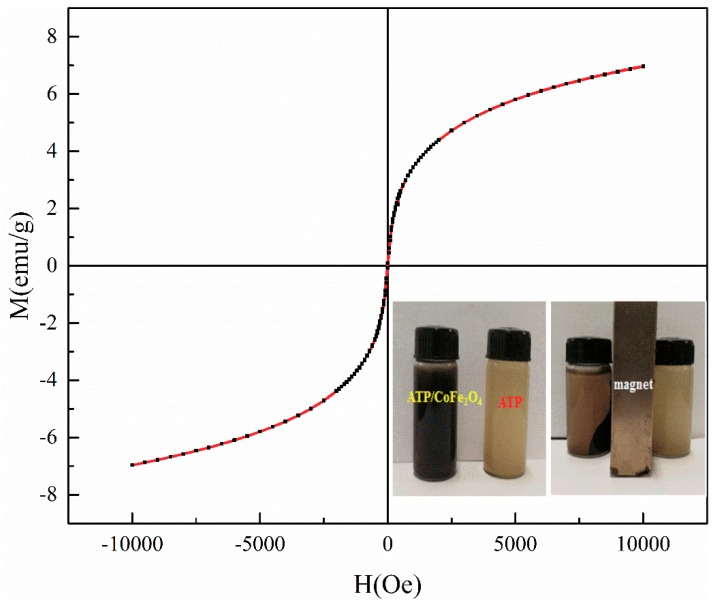
Vibrating sample magnetometry (VSM) analysis of ATP/CoFe_2_O_4_ magnetic composites.

Figure 4 shows the magnetic properties of ATP/CoFe_2_O_4_ investigated using VSM, and the ferromagnetic property is exhibited. The magnetization of ATP/CoFe_2_O_4_ is gradually increased and finally reaches equilibrium with increasing applied magnetic field. The magnetic field strength of the final ATP/CoFe_2_O_4_ also reaches equilibrium when a reverse magnetic field is applied. According to the calculation, the values of the hysteresis parameters, such as magnetization, remanent (Mr), and coercivity are 7 emu/g, 0.085 emu/g, and 8.059 Oe, respectively. The magnetic hysteresis loop of ATP/CoFe_2_O_4_ is an S-shaped curve, and no hysteresis occurs (Figure 4). The Mr is almost zero, and the curve almost passes through the origin. As can be seen from Figure 4, compared with ATP, the magnetic separation of ATP/CoFe_2_O_4_ is better, and ATP has almost no magnetic properties. Thus, ATP/CoFe_2_O_4_ possesses superparamagnetic properties, and solid–liquid separation can be effectively achieved under the condition of an external magnetic field [30]. 

### 3.2. TA Adsorption by ATP/CoFe_2_O_4_ Magnetic Composites

#### 3.2.1. Adsorption Kinetics 

**Figure 5:**
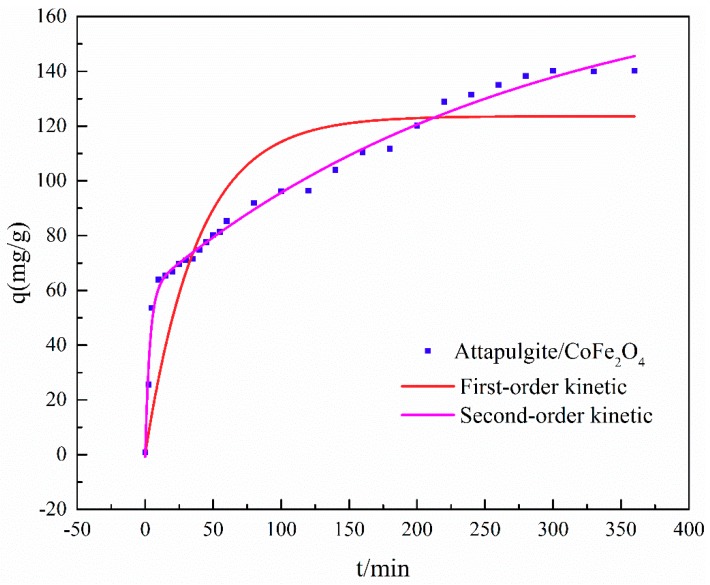
Adsorption kinetics of tannic acid (TA) on ATP/CoFe_2_O_4_ magnetic composites.

Figure 5 shows that the adsorption equilibrium for ATP/CoFe_2_O_4_ can be achieved within approximately 3 h for 1 L of 200 mg/L TA solution. In the first 40 min, the attapulgite/CoFe_2_O_4_ absorption capacity of TA increased rapidly to 75 mg/g. The TA adsorption capacity began to increase slowly within the time range of 40–180 min. The adsorption capacity from 180 min began to stabilize until adsorption equilibrium was reached at approximately 3 h.

There are two potential reasons for this change in adsorption capacity, one of which is contact time. The availability of the adsorption sites on ATP/CoFe_2_O_4_ and the TA concentration are high. In the first 40 min, these TA molecules are allowed to disperse rapidly, and they are easily captured by ATP/CoFe_2_O_4_. Upon prolonging the contact time (to within 40–180 min), an increasing number of adsorption sites are already occupied, and the TA concentration is reduced. Thus, the migration tendency of TA molecules to the surface of ATP/CoFe_2_O_4_ becomes low, which is not beneficial for the adsorption of other TA molecules. The adsorption capacity hardly increases with prolonged time (approximately 3 h) and remains constant until adsorption equilibrium [31]. The other reason is the peculiarity of the ATP/CoFe_2_O_4_ structure. The composition of CoFe_2_O_4_ on ATP/CoFe_2_O_4_ plays a dominant role in controlling the adsorption rate. CoFe_2_O_4_ can accelerate the mass transfer and diminish the diffusion limitation of the contaminant from aqueous solution to the surface of the adsorbent during adsorption, and the adsorption time to reach adsorption equilibrium is thus shortened [32].

The adsorption performance of TA on ATP/CoFe_2_O_4_ was fitted via the quasi-first-order kinetics and quasi-second-order kinetics. The values of the rate constants, *q* and *R*, are listed in Table 2. The kinetic model with a higher correlation coefficient (*R*) was selected as being more appropriate. As shown in Table 2, the high *R*^2^ value validates that the quasi-second-order kinetic model is suitable for describing TA adsorption on ATP/CoFe_2_O_4_.

#### 3.2.2. Adsorption Isotherms 

**Figure 6:**
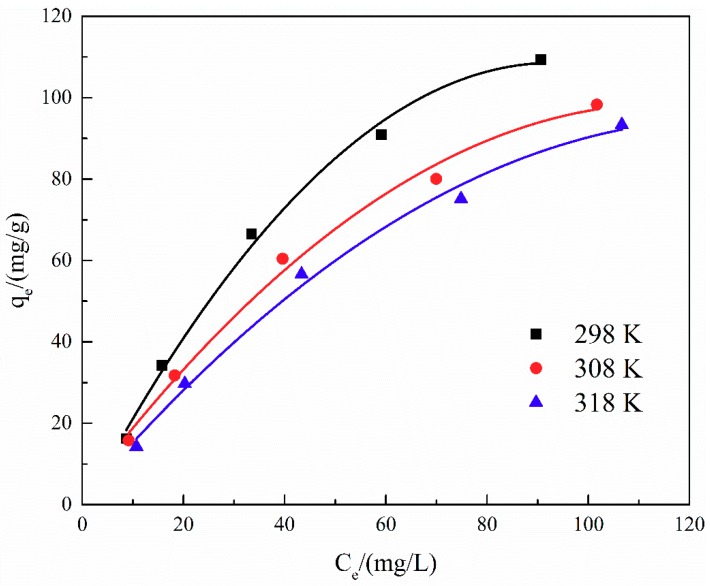
Adsorption isotherms of TA on ATP/CoFe_2_O_4_ magnetic composites.

**Figure 7:**
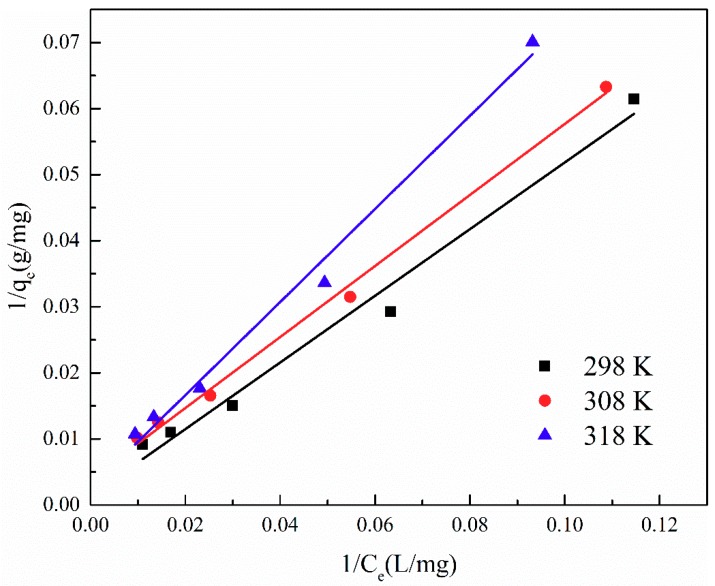
Fitting results of the Langmuir adsorption isotherm of TA on ATP/CoFe_2_O_4_.

**Figure 8:**
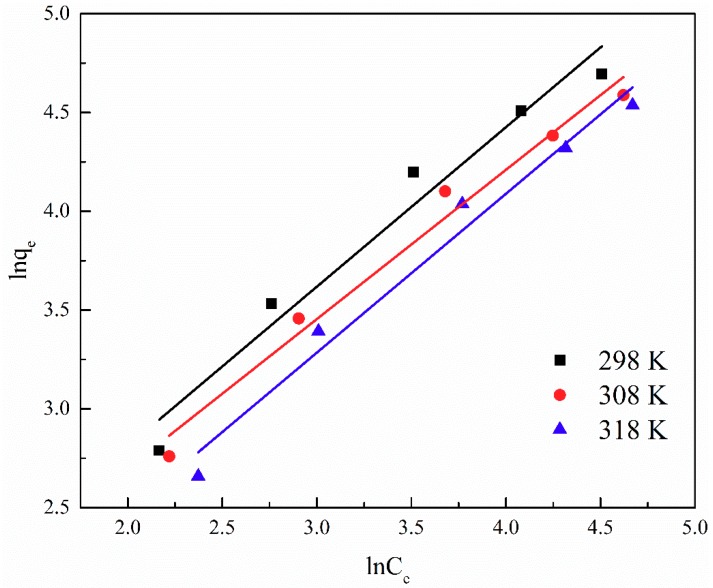
Fitting results of the Freundlich adsorption isotherm of TA on ATP/CoFe_2_O_4_.

The adsorption isotherm is important for describing the distribution of TA molecules between the liquid and the solid phases under an equilibrium state. Two different models (i.e., Langmuir and Freundlich) were employed to fit the isotherm data. The adsorption isotherms and the fitting results of ATP/CoFe_2_O_4_ for TA are shown in Figure 6, Figure 7 and Figure 8 and Table 3 and Table 4. Although the two models describe the experimental data, the equilibrium data of TA absorbed by ATP/CoFe_2_O_4_ are a better fit to the Langmuir adsorption isotherm than to the Freundlich adsorption isotherm based on a comparison of the *R*^2^ values (*R*_1_^2^ > *R*_2_^2^). The Langmuir isotherm assumes that monolayer adsorption is dominant. The adsorption capacity also decreases as temperature increases, demonstrating that adsorption is endothermic. The decrease in the adsorption equilibrium constant *k* with increasing temperature is caused by the gradual decrease in the adsorption capacity of ATP/CoFe_2_O_4_ as the temperature increases.

The effect of temperature on TA adsorption by ATP/CoFe_2_O_4_ was studied at 298 K, 308 K, and 318 K. The calculated values of these parameters are listed in Table 5. The results show that *G* is negative, indicating that TA adsorption on ATP/CoFe_2_O_4_ is thermodynamically feasible and spontaneous in nature. In addition, as *G*_0_ increases with temperature, low temperature can promote adsorption. The negative ΔS value shows that the degree of order of the adsorption system of ATP/CoFe_2_O_4_ increases. The value of ΔH is negative, indicating that TA adsorption on ATP/CoFe_2_O_4_ is exothermic [33].

### 3.3. Recyclability and Magnetic Performance of ATP/CoFe_2_O_4_ Magnetic Composites 

**Figure 9:**
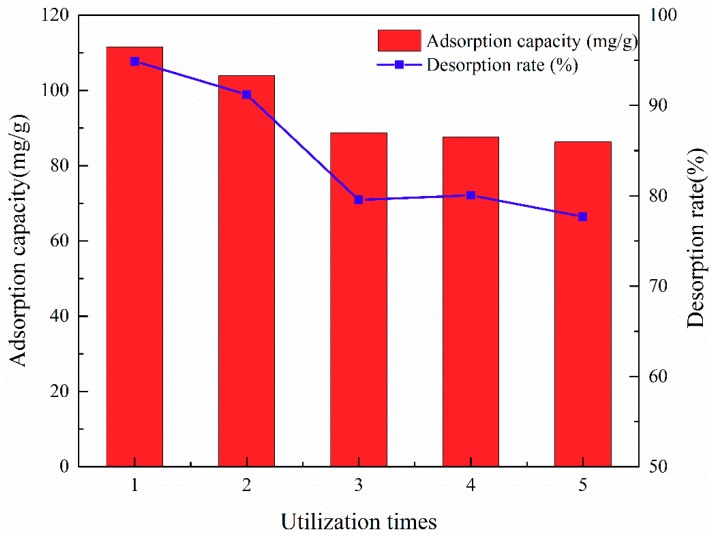
Reusability of the ATP/CoFe_2_O_4_ magnetic composites after five consecutive sorption–desorption cycles.

Given that an alkaline environment is not conducive to adsorption, 8% (w/w) NaOH solution was considered as the desorption liquid. The characteristics of ATP/CoFe_2_O_4_ allowed it to easily separate solid and liquid, so regeneration and circulation experiments were carried out. Figure 9 shows the recycling ability of ATP/CoFe_2_O_4_. For each cycle, the initial concentration of the TA solution was 200 mg/L. After five regeneration and cycling experiments, the concentration reached 90 mg/g although the adsorption capacity of ATP/CoFe_2_O_4_ decreased, and the desorption rate was stable at approximately 80%. Thus, ATP/CoFe_2_O_4_ has good regeneration ability and stability after NaOH solution desorption.

**Figure 10:**
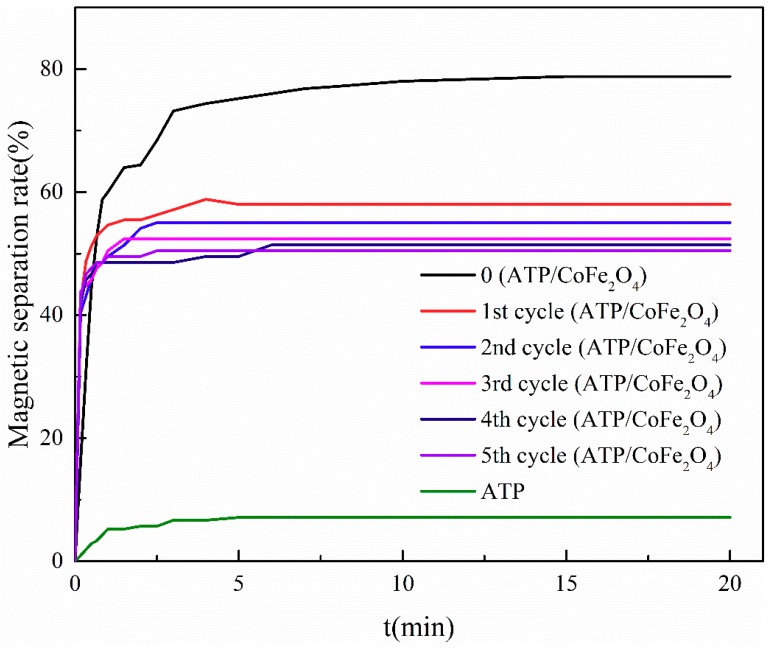
Magnetic properties of ATP/CoFe_2_O_4_ after five consecutive sorption–desorption cycles.

The changes in the magnetic separation performance of ATP/CoFe_2_O_4_ were investigated by observing the magnetic separation rate kinetic curves of ATP/CoFe_2_O_4_ (Figure 10). After the regeneration and circulation experiments, CoFe_2_O_4_ that did not tightly combine with ATP was desorbed by the NaOH solution. Therefore, the magnetic separation rate of ATP/CoFe_2_O_4_ declined after three cycles. The equilibrium time was prolonged gradually and stabilized at approximately 50%, indicating that CoFe_2_O_4_ was tightly combined on ATP, and the regeneration property of ATP/CoFe_2_O_4_ was good.

### 3.4. Removal Mechanisms of TA by ATP/CoFe_2_O_4_ Magnetic Composites

**Figure 11:**
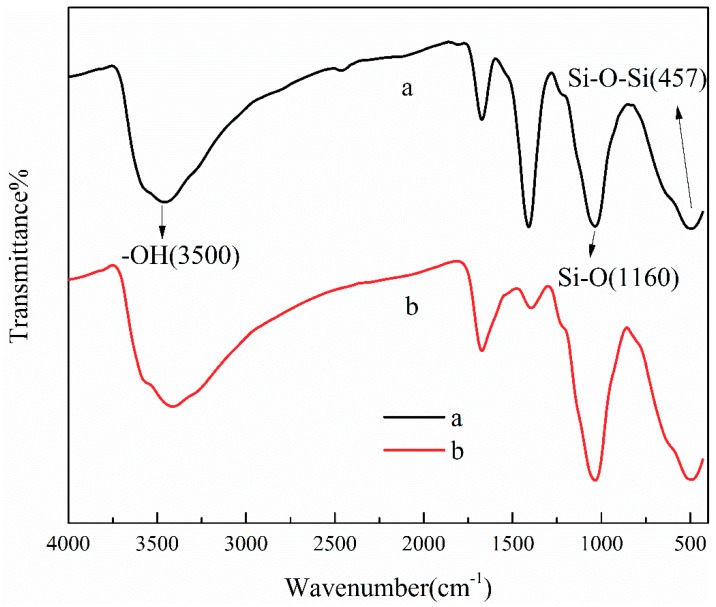
FTIR spectra of the ATP/CoFe_2_O_4_ magnetic composites before (**a**) and after TA adsorption (**b**).

The mechanisms of TA adsorption onto ATP/CoFe_2_O_4_ may be mainly attributed to intermolecular hydrogen bond formation and surface complexation [34,35], as shown in the FTIR spectra of ATP/CoFe_2_O_4_ before and after TA adsorption (Figure 11). The adsorption mechanisms of TA by ATP and CoFe_2_O_4_ are as follows.

As shown in Figure 11, the adsorption peak at approximately 3500 cm^−1^ evidently corresponds to the hydroxyl group [36]. After the adsorption of TA, the FTIR spectrum of ATP/CoFe_2_O_4_ exhibit certain changes. The relative intensity of this band obviously decreases. It broadens and shifts to decreased wavenumbers, revealing the important role of hydrogen bonding formation between TA and ATP in adsorption. One of the adsorption mechanisms of TA on ATP/CoFe_2_O_4_ may be that the hydroxyl group of ATP forms an intermolecular hydrogen bond with that of TA. When the hydroxyl groups are intermolecularly linked, a hydrogen-linked polymer is formed. The value of the bond force constant *K* decreases, so the absorption peak shifts to a lower wavenumber (approximately 3300 cm^−1^) [37]. The peaks at 1160, 1008, and 457 cm^−1^ intensify after adsorption. The silicon hydroxyl group formed by the cleavage of Si–O–Si on the surface of ATP has a certain adsorption capacity for organic pollutants such as TA [38].

Considering that intermolecular hydrogen bonds form, the surface complexation of the phenolic hydroxyl group (–OH) of TA and the ferric ion (Fe^3+^) on the surface of CoFe_2_O_4_ likely contributed to TA adsorption on ATP/CoFe_2_O_4_. These two functional groups can strengthen the interactions between ATP/CoFe_2_O_4_ and TA by forming surface complexes that contribute to high adsorption ability [39]. The complexation reactions can be divided into two steps: (1) dissociation of phenolic hydroxyl groups and (2) oxygen anions as a ligand for coordination with ferric iron on the CoFe_2_O_4_ surface.

Considering the molecular structure of TA, complexation usually occurs mainly in two ortho-positions of the phenolic hydroxyl groups due to the synergistic effect of two adjacent phenolic hydroxyl groups [40] and complexes with ferric iron to form a stable five-membered chelate ring structure, which makes CoFe_2_O_4_ adsorb TA to the surface.

## 4. Conclusions

ATP/CoFe_2_O_4_ magnetic composites were fabricated via a facile co-precipitation approach and employed to remove TA. The following conclusions were drawn:

(1) The morphology, structure, and magnetic properties of the synthesized composite were characterized using SEM, EDX, FTIR, XRD, and VSM. Multiple characterizations were conducted to illustrate that CoFe_2_O_4_ was successfully anchored on the surface of ATP during preparation.

(2) ATP/CoFe_2_O_4_ exhibited excellent adsorption capacity for TA of 109.36 mg/g. The adsorption equilibrium time of ATP/CoFe_2_O_4_ on TA was approximately 3 h. The adsorption kinetics of TA onto the composites followed the quasi-second-order kinetic model. The adsorption isotherm data were fitted well to the Langmuir isotherm.

(3) The adsorption–desorption studies showed that the adsorption capacity and desorption rate slightly decreased after five adsorption–desorption cycles. The composite adsorbent had high magnetic sensitivity under an external magnetic field, and its magnetic separation from water was allowed; secondary pollution was also avoided.

(4) Intermolecular hydrogen bonding formation and surface complexation were identified as the sorption mechanisms of TA by ATP/CoFe_2_O_4_.

Therefore, the magnetic composites combined the advantages of ATP and CoFe_2_O_4_, resulting in a fast-responsive adsorption rate, high TA adsorption capacity, and strong magnetic separation performance under an external magnetic field. This approach provides a novel and sustainable strategy for preparing magnetic ATP adsorbent.

## Figures and Tables

**Table 1:** EDX elemental analysis of CoFe_2_O_4_ and ATP/CoFe_2_O_4_.

Element	Weight Percentage		Atomic Percentage	
	CoFe_2_O_4_	ATP/CoFe_2_O_4_	CoFe_2_O_4_	ATP/CoFe_2_O_4_
C	15.99	24.25	24.64	36.81
N	11.26	/	14.88	/
O	38.66	41.61	44.71	47.42
Na	9.77	2.67	7.86	2.11
Mg	/	1.71	/	1.28
Al	/	1.16	/	0.79
Si	/	7.2	/	4.67
Ca	/	0.41	/	0.19
Fe	8.46	13.77	5.25	4.49
Co	15.85	7.22	2.66	2.23
Total	100	100		

EDX: Energy dispersive X-Ray spectroscopy.

**Table 2:** Kinetic parameters of TA adsorption on ATP/CoFe_2_O_4_ magnetic composites.

First-Order Kinetics			Second-Order Kinetics			
$R_{1}^{2}$	$q_{1}$	$k_{1}$	$R_{2}^{2}$	$q_{2}$	$k_{2}$	*h*
0.6873	211.8122	0.0215	0.9779	147.0588	0.0002071	4.4783

**Table 3:** Langmuir constants for TA adsorption on ATP/CoFe_2_O_4_ magnetic composites.

T/K	Langmuir			
	Equation	$R_{1}^{2}$	$q_{m}$	*B*
298	1/*q*_e_ = 0.5045/C_e_ + 0.0014	0.9841	714.2857	0.0028
308	1/*q*_e_ = 0.5375/C_e_ + 0.0039	0.9964	256.4103	0.0073
318	1/*q*_e_ = 0.7046/C_e_ + 0.0025	0.9906	400.0000	0.0035

**Table 4:** Freundlich constants for TA adsorption on ATP/CoFe_2_O_4_ magnetic composites.

T/K	Freundlich			
	Equation	*R*_2_^2^	*k*	*n*
298	ln *q*_e_ = 0.8073ln *C*_e_ + 1.1963	0.9656	3.3079	1.2387
308	ln *q*_e_ = 0.7554ln *C*_e_ + 1.1881	0.9806	3.2808	1.3238
318	ln *q*_e_ = 0.8039ln *C*_e_ + 0.8727	0.9778	2.3934	1.2439

**Table 5:** Kinetic parameters of TA adsorption on attapulgite/CoFe_2_O_4_ magnetic composites.

T/K	ΔH(KJ/mol)	ΔS(J/(mol·K))	ΔG(KJ/mol)
298	−12.61	−31.95	−2.96
308			−3.04
318			−2.31
